# Suppress or Not to Suppress … CRAFT It: A Targeted Metabolomics Case Study Extracting Essential Biomarker Signals Directly from the Full ^1^H NMR Spectra of Horse Serum Samples

**DOI:** 10.3390/metabo15060387

**Published:** 2025-06-10

**Authors:** James Chen, Ayelet Yablon, Christina Metaxas, Matheus Guedin, Joseph Hu, Kenith Conover, Merrill Simpson, Sarah L. Ralston, Krish Krishnamurthy, István Pelczer

**Affiliations:** 1 LLP 2024, Department of Chemistry, Princeton University, Princeton, NJ 08544, USA; jztc21@gmail.com (J.C.); christina.amet@gmail.com (C.M.); mguedin@outlook.com (M.G.); joseph.hu.44@gmail.com (J.H.); 2 Department of Chemistry, Princeton University, Princeton, NJ 08544, USA; kenithc@princeton.edu; 3 Department of Animal Science, Rutgers University, New Brunswick, NJ 08544, USA; 4 Independent Researcher, Howell, NJ 08544, USA; ralstonvmd@msn.com; 5 Chempacker LLC, San Jose, CA 95135, USA; chempacker@gmail.com

**Keywords:** biomarkers, targeted metabolomics, NMR, signal processing, CRAFT, inflammation, horses

## Abstract

**Background**: There are a few very specific inflammation biomarkers in blood, namely lipoprotein NMe^+^ signals of protein clusters (GlycA and GlycB) and a composite resonance of phospholipids (SPC). The relative integrals of these resonances provide clear indication of the unique metabolic changes associated with disease, specifically inflammatory conditions, often related to serious diseases such as cancer or COVID-19 infection. Relatively complicated, yet very efficient experimental methods have been introduced recently (DIRE, JEDI) to suppress the rest of the spectrum, thus allowing measurement of these integrals of interest. **Methods**: In this study, we introduce a simple alternative processing method using CRAFT (Complete Reduction to Amplitude-Frequency Table), a time-domain (FID) analysis tool which can highlight selected subsets of the spectrum by choice for quantitative analysis. The output of this approach is a direct, spreadsheet-based representation of the required peak amplitude (integral) values, ready for comparative analysis, completely avoiding all the convectional data processing and manipulation steps. The significant advantage of this alternative method is that it only needs a simple water-suppressed 1D spectrum with no further experimental manipulation whatsoever. In addition, there are no pre/post processing steps (such as baseline and/or phase), further minimizing potential dependency on subjective decisions by the user and providing an opportunity to automate the entire process. **Results**: We applied this methodology to horse serum samples to follow the presence of inflammation for cohorts with or without OCD (Osteochondritis Dissecans) conditions and find diagnostic separation of the of the cohorts through statistical methods. **Conclusions**: The powerful and simple CRAFT-based approach is suitable to extract selected biomarker information from complex NMR spectra and can be similarly applied to any other biofluid from any source or sample, also retrospectively. There is a potential to extend such a simple analysis to other, previously identified relevant markers as well.

## 1. Introduction

Metabolomics and metabonomics—these terms recently converged to a consensus metabolomics term—are the study of molecules, known as metabolites, found within biological systems [1,2,3]. Metabolites are the molecules produced or consumed during the chemical reactions that occur in the body to sustain life. The sum of all metabolites at any given time is called the metabolome, and it displays the chemical state of the body and provides information about nutrition, health, and disease status [3]. The spectral data-generated NMR and/or MS represents the full range of metabolites present in a biofluid [4].

Biofluids, such as blood serum or plasma, are natural targets for NMR-based metabolomics, including quantitative assessment of selected components in the mixture. There are a small number of such component resonances, namely N^+^Me signals of lipoproteins (GlycA and GlycB [5]), and a composite signal of phospho-glycoproteins (SPC [6]), which are especially indicative of inflammatory conditions. For data analysis the most commonly used multivariate statistical tool is O-PLS-DA [6].

There are sophisticated experimental methods introduced recently (DIRE [7] and JEDI [8]) to suppress practically all the unwanted signals from the complicated ^1^H NMR spectra of serum or plasma samples retaining the signals of essential inflammatory biomarkers. However, such experiments are technically demanding, they lead to the overall decrease of sensitivity, and the suppression process may have uncontrolled influence on the signals of interest, as well. In addition, conventional NMR processing methods to equate signal integrals and/or height to relative concentration have dependencies on tedious and subjective phase/baseline corrections as well as peak-resolution and noise-dependent integration uncertainty [9].

**Figure 1:**
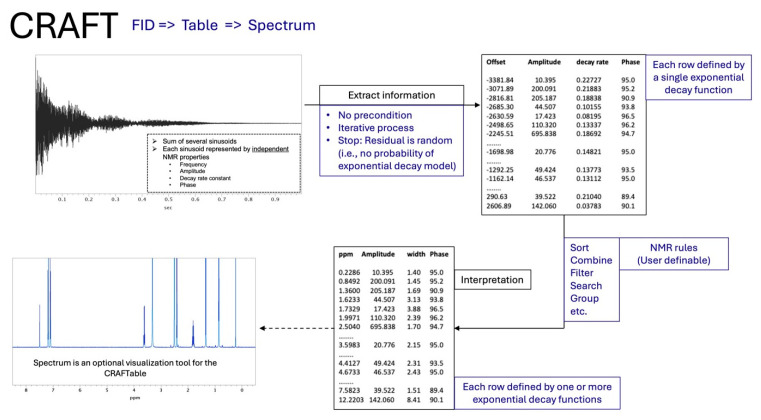
Schematic summary of the CRAFT protocol.

As a simple and efficient alternative, we instead turned to the powerful software tool, CRAFT (Complete Reduction to Amplitude-Frequency Table) [10], which directly analyzes the time domain NMR data (FID) with no additional manipulation or data treatment, and presents the content in a spreadsheet format, including the amplitude for quantitation, as opposed to traditionally analyzed frequency domain spectra (Figure 1).

This technique eliminates the need for phase and baseline correction, therefore minimizing errors. Furthermore, CRAFT produces more reliable amplitudes of overlapping signals compared to frequency-domain analysis. CRAFT has been shown to be very powerful for analysis of complex mixtures [10], even applied to two-dimensional spectra [11], also in combination with non-uniform sampling [12]. A comprehensive mini-review and references therein summarize the capabilities and many typical applications of CRAFT [13]. It is important to point out that CRAFT is a workflow and not a hidden/compiled code, therefore, there are no formal releases, nor a is no specific version number. The actual interactive interface has been, and is being constantly modified and enhanced by the author, K.K. All the details of this methodology/workflow can be found in peer-reviewed papers, and we expect the citations provided are sufficient for the reader’s reference. The disparate elements required to put together the workflow are available freely or in various available NMR software. This is specifically explained in the published papers and the review. A package of such workflow is also commercially available and may save the reader some effort.

**Figure 2:**
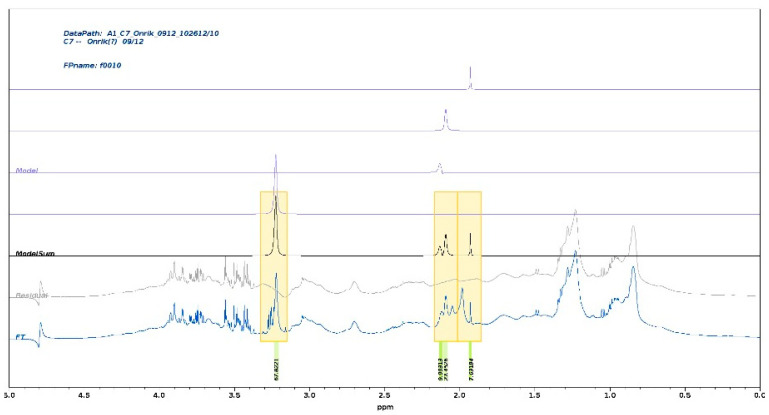
A stacked plot of conventional frequency domain spectra and CRAFT models of an arbitrarily selected spectrum from the set of 40 spectra—only the upfield region is shown. The selections for CRAFT modeling are highlighted with the color boxes, including the acetate methyl resonance at 1.92 ppm. The bottom trace is the conventional spectrum; the following traces upwards are the same spectrum when the CRAFT model is subtracted for the full data, the complete CRAFT model, followed by the four traces of the individual selected components, respectively. The a.u. amplitudes are shown below the plot.

Our approach uses the full 1D spectrum (only water suppression applied). Therefore, all resonances will be present with undisturbed intensity. Partial overlap with other signals or broad background resonances can be efficiently handled (see Figure 2). We have been exploring the various strategies of parametrization in CRAFT, tested diffusion-filtered spectra to assess the effect of experimental signal suppression and compared results of tedious conventional integration (line fit) methods with CRAFT data with close match.

We applied the CRAFT protocol to 20 + 20 horse serum samples from healthy and diseased subjects diagnosed with OCD, respectively, as a case study and a proof of concept. All data processing was accomplished by the group of five high school students who joined the summer LLP program. LLP stands for Laboratory Learning Program, designed for senior high school students to join actual research at Princeton University for a few weeks in the summer. Faculty can offer program projects which are evaluated and ranked. Typically, there are dozens of student applications for a few seats for each program.

Osteochondrosis dissecans in horses is a developmental orthopedic metabolic defect, essentially a metabolic disease which has possible genetic contribution and a significant inflammatory aspect [14] caused by abnormal ossification of cartilage at the growth plates [15,16]. A weakened cartilage–bone matrix that is under stress may form cartilage lesions within the articular space, potentially reducing the performance ability of affected horses. Osteochondrotic lesions appear during the first year of life in rapidly growing horses, but clinical signs may not appear until the horse is put into training at two or three years of age.

Osteochondrosis can be induced or exacerbated by factors such as improper nutrition, exercise, and trauma [16,17]. A correlation between the presence of OCD lesions and abnormal glucose and insulin metabolism has been reported [18,19], but cause/effect relationships have not been documented. Osteochondrosis is particularly prevalent in certain breeds of horses, such as Standardbreds, Thoroughbreds, and Quarterhorses [15,17,20], with genetic predisposition well established in Standardbreds [20], but the specific metabolic defects have not been identified. Several genes were found that contain quantitative trait loci for Osteochondrosis, signifying heritability of OCD in the tibiotarsal joint of Norwegian Standardbred trotters [21]. A mixed-model statistical analysis revealed seven significant single-nucleotide polymorphisms (SNPs) that showed moderate correlation with OCD status [21]. Mixed-model statistics contain both fixed and random effects, which are used to refer to population-average and individual-specific effects [21,22].

We applied selective CRAFT extraction of the representative inflammatory markers [5,6]. The amplitude ratios of the Gly and SPC signals clearly identify the diseased cohort with good validation in O-PLS-DA. Similar analyses of multiple datasets from a variety of earlier studies are in progress, too.

## 2. Materials and Methods

### 2.1. Sample Preparation, Data Acquisition

For this case study, we used a cohort of 20 + 20 horse samples, healthy and OCD diagnosed individuals, respectively, collected in 2012 [23]. The OCD condition is commonly identified by radiography, as it was for these horses as well. The metabolic analysis has the great potential advantage that it may reveal the developing condition in its early phase when radiography can fail or can be ambiguous. Sometimes, this diagnosis takes place later, then the ametabolic information can be cross-validated with the results. The special advantage of the CRAFT analysis is that it can be done retrospectively on any data acquired earlier, as the water-suppressed full spectrum is naturally part of the typical acquisition package [24].

Serum samples were collected from 40 young Standardbred horses from Hanover Shoe Farm in Hanover, PA, in August 2011. The samples were from 20 matched pairs of yearling Standardbred horses that had been paired based on lineage, each member of the pair having the same sire and similarly bred dams, to reduce additional sources of variations. The only difference between the two horses in each pair was that one had had hock OCD lesions, the other determined by radiographs to be unaffected (control). Each serum sample was separated into 4 aliquots and stored in −80 °C. The samples were submitted to NMR analysis.

The ^1^H NMR spectra of these serum samples were run on a Bruker 500 MHz spectrometer with a TCI CyroProbe and large capacity BACS-120 autosampler designed for high efficiency small molecule studies (Bruker-Biospin, Billerica, MA, USA). For the experiments, we routinely use a concentric tube construct with a 4 mm OD insert carrying the actual sample, inserted into a 5 mm OD container tube. The sample is not modified in any way, while a small amount of D_2_O is added to the container tube filling the gap between the tubes and providing the lock signal. For every sample, we conduct individually controlled shimming and pulse calibration. Typical acquisition parameters are 32 k data points over 20 ppm window, 64 scans for each spectrum, 5 s overall recycle delay, 298.2 K controlled temperature.

We routinely collect four spectra on the horse serum samples following standard procedures [24]: full spectrum with water suppression only (we prefer Excitation Sculpting [25] for the water suppression); relaxation filtered spectrum using a long spin-l0 ck (SL) pulse; diffusion filtered spectrum; and a 2D-J spectrum. It is worth noting that there exist sophisticated computation-based methods, such as SMolESY [26], to suppress large molecule contributions with the purpose to highlight the small molecule content. However, it is important to note that our discussion is not about this type of separation or classification, which rather relates to the relaxation filtering in experiment. The experimental approaches we refer to in comparison (DIRE [7] and JEDI [8]) rely on selection of the signals of interest based on diffusion, relaxation, and J-filtering in combination. Our CRAFT-based approach bypasses all these and extracts the signals of interest by choice.

Excitation sculpting is one of the two major techniques used in metabolomics for water suppression; the popular alternative is 1DNOESpr. The latter one is clearly more robust and easier to set up for automation; however, it has a specific drawback that resonances of exchanging moieties can be partially suppressed in an uncontrolled fashion. NOE effects throughout large molecules (proteins, lipoproteins) may also modulate signal intensities—as we saw it in test experiments. On occasion, we do use 1D NOESYpr, but most of the time, as in this study, ES is our primary choice for more efficient suppression and avoiding the effects mentioned above. For each experiment, we carefully calibrate the pulses and set up the experiment manually, one at a time. In this current study, only the full spectrum results are discussed, as the CRAFT extraction is readily capable to access and selectively analyze the signals of interest, without need for any experimental suppression of unwanted signals.

### 2.2. Data Processing, Multivariate Statistical Analysis

For conventional data processing to produce frequency domain data for untargeted multivariate statistical analysis, the usual, standard protocol was applied, readily available in the MestreNova software. Apodization, phase correction, baseline correction, calibration, global and local peak alignment were applied as described [23,27]. The alpha-glucose alpha-H doublet was set to 5.233 ppm as reference for calibration [28]. Conventional NMR spectral data processing and visualization were conducted using MNova, up to v.14.3 (MestreLab Research S.L., Santiago de Compostela, Spain). We wish to point out again that the CRAFT protocol avoids all these steps, uses only and exclusively the native time domain data (FID), and extracts the relevant spectral parameters directly.

Multivariate statistical analysis was done in SIMCA (v.16, Sartorius/Umetrics, Umea, Sweden). In preparation for the multivariate analysis of the conventional data, the processed spectra were exported to Excel, and the full set was saved as a csv output file, which is accepted in SIMCA. Various models were calculated and carefully validated, where the O-PLS-DA models are most informative [6]. Distinct clustering on a scores scatter plot is indicative of differences between OCD and control groups. Validity of the statistical models was tested using the R^2^ and Q^2^ values, R^2^ representing the goodness of fit and Q^2^ representing the predictability [6]. Cross-validation is completed by removing data elements from the model and re-inserting them to be compared to the R^2^ and Q^2^ values of the original model, a standard procedure in the SIMCA software. The original model R^2^ and Q^2^ values should be higher than the cross-validated R^2^ and Q^2^ values [6]. All data presented here were mean centered, and univariate scaling was used.

All targeted CRAFT time-domain NMR data processing [10,13] was done using the commercially available software developed by K. K. (Chempacker LLC, San Jose, CA, USA). Output amplitude data were saved immediately in an online Excel file, which was continuously inspected and corrected, as needed, by the LLP students. The identified amplitude values were then used as the input for O-PLS-DA analysis in SIMCA. The five LLP students have worked closely as a team, shared and compared actual intermediate results; the exemplary interactive, real-time collaboration was essential for the whole procedure.

## 3. Results and Discussion

**Figure 3:**
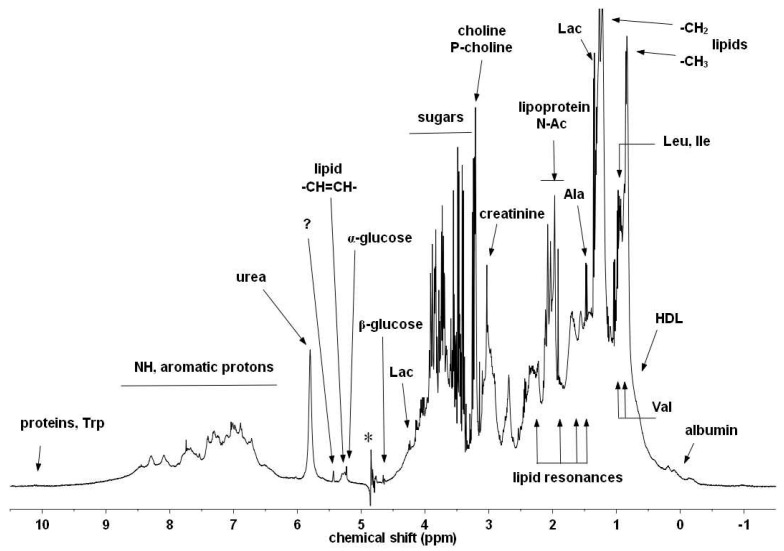
Typical ^1^H-NMR spectrum of horse serum. Some of the characteristic metabolites and other components are denoted [29] (with permission). The asterisk labels the residual artefact of the suppressed water signal.

A typical 1D spectrum of a horse serum sample with only ES water suppression [25] is shown with annotations for selected components (Figure 3). It is a complicated mixture of small molecules and large proteins, lipids, and lipoproteins.

**Figure 4:**
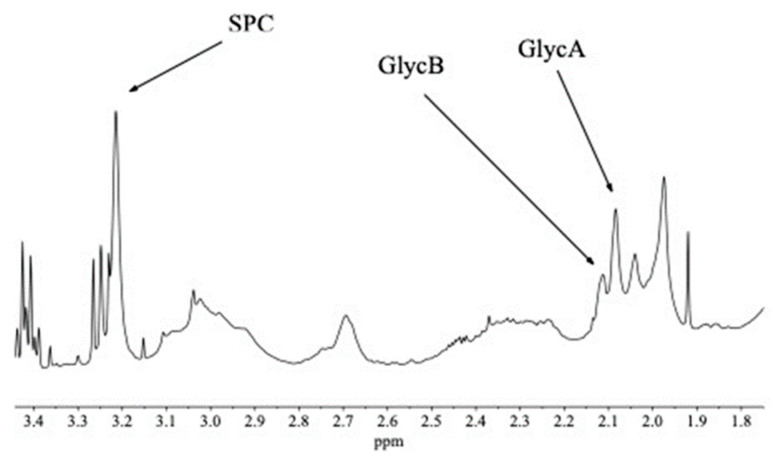
Expanded section of a randomly selected full spectrum using ES water suppression only. This section of the spectrum contains many resonances which belong to small molecules, such as the sharp peaks of geta-glucose at ca. 3.42 and 3.25 ppm, respectively, and the singlet at 1.92 ppm, the acetate methyl resonance, as well as broad resonances, mostly belonging to unsaturated and saturated lipids and lipoproteins. The three inflammatory biomarker resonances are highlighted.

In the full spectrum (Figure 3) there is significant overlap for these three signals of interest with other peaks—beta-glucose (for SPC), broad resonances of unsaturated lipids (for GlycA and GlycB), respectively (see Figure 4). Instead of using experimental suppression methods, our CRAFT approach handles the data, the time domain FID signal only, and extracts the relevant parameters for the signals of interest directly, while avoiding all conventional data processing steps (apodization, FT, phase- and baseline-correction, etc.).

### 3.1. CRAFT Data Analysis

An expanded section of a randomly selected full spectrum from the set of 40 samples is shown in Figure 4. The three peaks of interest to us, which are the biomarkers for inflammation (SPC, GycB, GlycA), are annotated.

A representative CRAFT analysis (see Figure 2) presents illustrative frequency domain representation of the CRAFT models. The CRAFT model was generated extracting the SPC, GlycB, and GlycB peaks, as well as the acetate methyl resonance at 1.92 ppm as an added control. The SPC signal is cleanly separated from the doublet-of-doublet resonance of the beta-glucose at 3.21 ppm. In the cluster at about 3 ppm, the GlycB, GlycA, and the acetate methyl singlet are modeled individually.

CRAFT is a time domain analysis which filters the selected frequency segments, ignoring the first few points in the FID, back-calculating to the t = 0, thus avoiding baseline and phase correction and providing the correct, quantitative amplitude of the selected peak or peaks. This is key for the analysis of the inflammatory biomarkers.

### 3.2. Multivariate Analysis of Selected Integral Ratios

The amplitudes of the three biomarker resonances carry information which can be used for diagnostic distinction of diseased and healthy subjects. As the excellent review of Fuertes-Martín and co-workers [5] reports, the GlycA/GlycB integral ratio has been identified in dozens of research projects to correlate with various inflammatory conditions and disease states. The complex SPC resonance was found to be characteristically correlated with the GlycB showing strong indication of metabolic disturbances. More detailed studies [30], including most recent population studies [31], found subcomponents (SPC1, SPC2, SPC3), which provided more insight into these conditions. At low field (80 MHz), diagnostic analysis is still possible after applying the JEDI signal suppression, but these components are not resolved [32].

**Figure 5:**
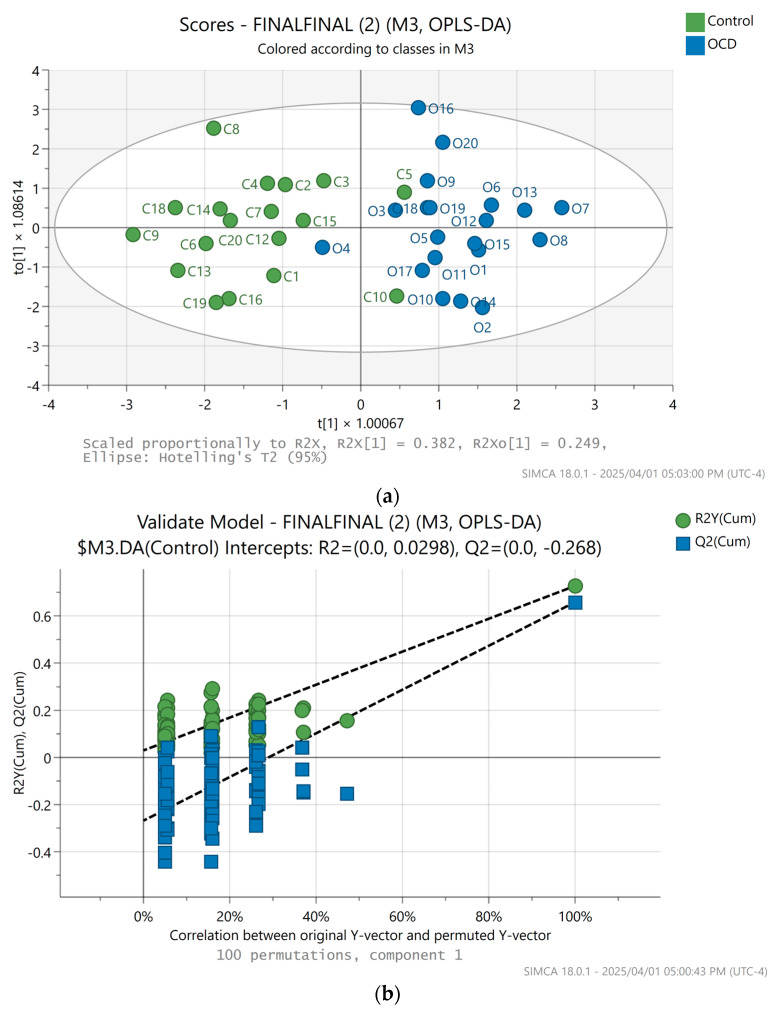
(**a**) SIMCA multivariate analysis result, O-PLS-DA scores plot of the SPC/GlycB amplitude ratios. Blue dots represent horses with OCD diagnosis, while the green dots belong to the healthy horses. For this calculation, C11, C17 were excluded, two outliers. (**b**) SIMCA multivariate analysis permutation plot result, validation of the O-PLS-DA scores plot of the SPC/GlycB amplitude ratios.

In our study, we checked the various ratios of the amplitudes of the three extracted biomarkers, submitting those for multivariate analysis in SIMCA. The O-PLS-DA scatter plot [6] of the GlycB/SPC ratio showed the best clustering, with high-quality validation clearly separating the horses with OCD from the healthy cohort (Figure 5a,b).

**Figure 6:**
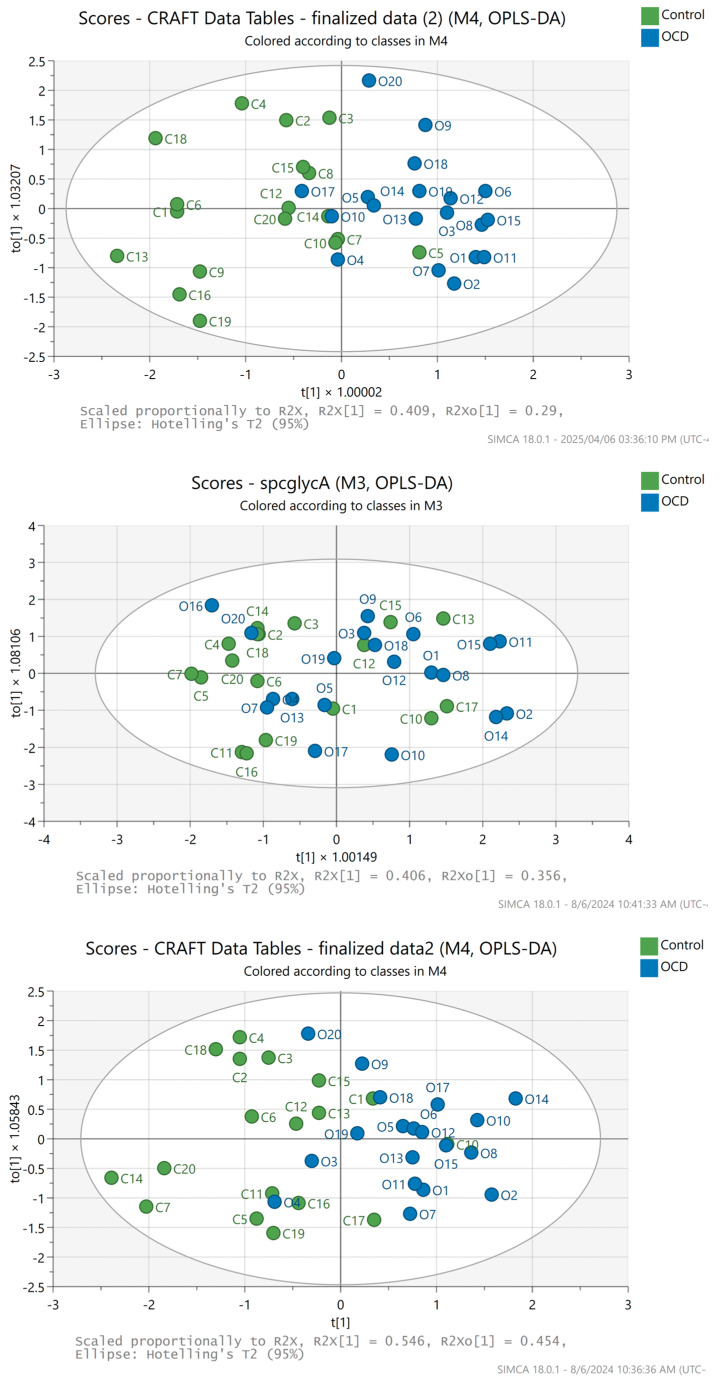
SIMCA multivariate analysis results; O-PLS-DA scores plot of the GlycA/GlycB (**top**), where C11, C17, and O16 were excluded (3 outliers); SPC/GlycA (**middle**), where C8 and C9 were excluded (two outliers); SPC/(GlycA + GlycB) (**bottom**) amplitude ratios, where C8, C9, and O16 were excluded (3 outliers), respectively. Blue dots represent horses with OCD diagnosis, while the green dots belong to the healthy horses.

None of the other combinations of ratios were as satisfactory (Figure 6), although the GlycA/GlycB O-PLS-DA score plot and validation results are the second in line for good separation and quality, respectively, agreeing with the findings in the review [5].

## 4. Summary, Conclusions, Future Work

We have applied the CRAFT [10,13] software tool to extract essential inflammatory biomarker signals quantitatively using only the full, water-suppressed 1D ^1^H spectrum of horse serum samples. This simple approach is a competitive alternative to a variety of sophisticated experimental methods which aim to suppress the rest of the spectrum and keep only the peaks of interest [6,8]. The CRAFT selective extraction uses the full information content of the spectrum without any modifications or loss of sensitivity. GlycA, GlycB, and SPC can be quantitatively extracted from complex spectral environments, including overlap with other, irrelevant signals and broad background resonances.

The most important message is that for targeted analysis, extracting relevant parameters (in this case primarily the amplitude) for selected resonances is a viable and highly efficient alternative to the conventional full-processing protocol followed by untargeted multivariate statistics. Indeed, we extract three signals only, which are identified as characteristic inflammatory markers. This is truly a limitation compared to the full information content of the untargeted analysis, but this is the very nature of the targeted process. With the CRAFT analysis of these three signals, we managed to separate and identify the healthy and OCD cohorts very quickly and efficiently and proved the inflammatory component of the condition. Obviously, the CRAFT extraction does not replace the experimental suppression approaches, but rather offers an orthogonal, quick, and very economical diagnostic approach. In general, quantitative CRAFT analysis can be applied to any other identified biomarkers or their patterns.

In this work, we proved that CRAFT is a viable and highly simplified tool for diagnostics from a single and simple spectrum. This work focuses on the three major inflammatory biomarker signals (GlycA, GlycB [5], and SPC [6]), but the concept is valid to all identifiable biomarker signals or patterns of those. The recent implementation of the lipoprotein subclass analysis on low-field benchtop instruments (FT-80 from Bruker [33]) is a very competitive alternative for direct clinical applications. It remains an interesting question whether the CRAFT analysis could be useful for those low-dispersion spectra.

This work proves that OCD, which is a complex metabolic disorder, has a contributing inflammatory component indeed, as the ratio of the GlycB and SPC amplitudes clearly separate the healthy and diseased cohorts using multivariate analysis, as shown by the O-PLS-DA results with very good validation. Other combinations of the selected signal amplitudes show somewhat limited or no good clustering.

It is an extremely beneficial feature of the CRAFT analysis, that it can be applied retrospectively on any relevant data collected in the past.

We are in the process of extending this application to many other datasets collected earlier, including those on laminitis, age related studies, comparing metabolic signature of mustangs to that of domestic horses, falcon aspergillosis, etc.

## Data Availability

The data presented in this study are available on request from the corresponding author.

## Abbreviations

The following abbreviations are used in this manuscript:

LLP	Laboratory Learning Program, Princeton University, 2024
SPC	composite resonance of phospholipids
OCD	Osteochondritis Dissecans
CRAFT	Complete Reduction to Amplitude-Frequency Table
FID	Free induction decay
